# Immune Checkpoints Inhibitors and SRS/SBRT Synergy in Metastatic Non-Small-Cell Lung Cancer and Melanoma: A Systematic Review

**DOI:** 10.3390/ijms222111621

**Published:** 2021-10-27

**Authors:** María Rodríguez Plá, Diego Dualde Beltrán, Eduardo Ferrer Albiach

**Affiliations:** Department of Radiation Oncology, Hospital Clinico Universitario de Valencia, 46010 Valencia, Spain; dualde_die@gva.es (D.D.B.); ferrer_edu@gva.es (E.F.A.)

**Keywords:** stereotactic radiosurgery, stereotactic body radiation therapy, immune checkpoint inhibitors, radiation therapy, anti-PD-L1, anti-CTLA4, ICI-SBRT, ICI-SRS

## Abstract

*Background:* Several immunotherapy (IT) agents are FDA approved for treatment of melanoma and non-small-cell lung cancer (NSCLC). The addition of stereotactic radiosurgery (SRS) or stereotactic body radiation therapy (SBRT) to immunotherapy looks promising. A systematic review was conducted to evaluate the possible synergistic effects of immune checkpoints inhibitors (ICIs) and stereotactic radiation therapy in melanoma and NSCLC. *Materials and methods:* Pubmed databases from January 2010 to December 2020 were reviewed to identify English language studies reporting control of local and abscopal effect of the combination of ICI-SBRT/SRS in metastatic NSCLC and melanoma cancer. The inclusion criteria were followed according to PICO criteria. *Results:* Thirty-nine articles were included of the 2141 initial results. The reported rates for local control were 16.5–100% and 40–94% in brain and extracerebral metastases, respectively. Distant/abscopal response rates were 1–45% in extracerebral metastases. Abscopal effect could not be evaluated in brain metastases because it was not reported in studies. Treatments were well tolerated with few grade 4 toxicities and no grade 5. *Conclusions:* The combined treatment of ICI-SBRT/SRS achieves high local control and non-negligible abscopal response in patients with extracerebral metastases, with its benefit in cerebral metastases being more controversial. Clinical trials are needed to better characterize the potential synergism.

## 1. Introduction

Local radiation therapy (RT) is known to modulate the immune response [1,2]. The development of immunotherapeutic drugs has led to a rapid growth of publications that have attempted to elucidate the potential synergistic effects of the combined treatment of immunotherapy with radiation therapy. This combination is known as immunoradiotherapy [3,4].

It has been shown that combined immunoradiotherapy treatment can promote greater local control and an antitumor systemic response (response known in the literature as the abscopal effect) through T cell-mediated activation of the adaptive immune system [5,6]. Radiation therapy treatment induces a stress response or cell death by stimulating the production of tumor-associated antigens that activate antigen-presenting cells (APCs). Activation of APCs induces activation of specific CD8+T lymphocytes against tumor cells presenting these antigens. These circulating activated lymphocytes can be extravasated to non-irradiated tumor lesions, and can act on them [2,7].

Stereotactic radiosurgery and stereotactic body radiation therapy have undergone exponential development in recent years, as their ablative capacity has demonstrated a benefit in certain patients, such as oligometastatic or oligoprogressive patients [8]. Oligoprogression is a limited tumor progression with the rest of the disease controlled. Under ICIs, oligoprogression occurs in approximately 10–20% of cases [9]. In these patients, SRS/SBRT allows the administration of a high antitumor biologically effective dose (BED). Different fractions are used according to the anatomical location, size, and tumor histology, among other factors. In general, the most frequent fractioning schemes used in SRS/SBRT are those in which a dose per fraction >6 Gy is administered, in 1–5 fractions.

Currently, it is unknown which doses per fraction obtain a greater antitumoral immune response. However, preclinical models have shown that doses between 10 and 13 Gy seem to maximize these effects [7]. In addition, it has been shown that SBRT promotes a signaling cascade secondary to the destruction of the tumor stroma that promotes immune-mediated tumor recognition [10]. SRS/SBRT decreases repair of sublethal damage and tumor repopulation.

On the other hand, several immunotherapies are FDA approved for treatment of melanoma and NSCLC. ICIs including anti-cytotoxic T lymphocyte-associated protein 4 (CTLA4) and anti-programmed death-1 (PD1) antibodies have become the most widely used agents in this field. ICIs act by blocking checkpoint proteins from binding with their partner proteins.

T cells recognize antigens presented by the major histocompatibility complex (MHC) on the surface of cancer cells through their T cell receptor (TCR). This first signal is not sufficient to turn on a T cell response, and a second costimulatory signal consisting of the union B7 (CD80 or CD86) and CD28 is required. CTLA4 is present in the membrane of T cells, especially in regulatory T cells. It inhibits the costimulatory signal needed for T cell activation by competing with CD28 for binding B7. Anti-CTLA4 is a monoclonal antibody that acts by inhibiting CTLA4 and consequently stimulates T cell activation after antigen presentation [11].

PD-1 is expressed on activated T cells and mediates inhibitory signals upon binding to its ligand PD-L1, which is expressed on tumor cells and antigen-presenting cells. Its blockade with PD-1 or PD-L1 antibodies results in the activation of T cells against tumor cells [11].

Immunotherapies can also be used to enhance immune responses together with SBRT because both increase T cell activation and reduce cancer immune evasion [12].

Moreover, the apparent synergy between ICI and radiotherapy is potentially useful not only in stage IV tumors with oligoprogression but also with, e.g., stage III NSCLC patients not eligible for chemotherapy, who could receive immunoradiotherapy instead of the standard chemoradiotherapy [13].

Knowing the benefits of SBRT/SRS combined with ICIs and possible side effects, as well as the best sequence or timing of treatment, are key to our daily practice.

## 2. Results

**Figure 1:**
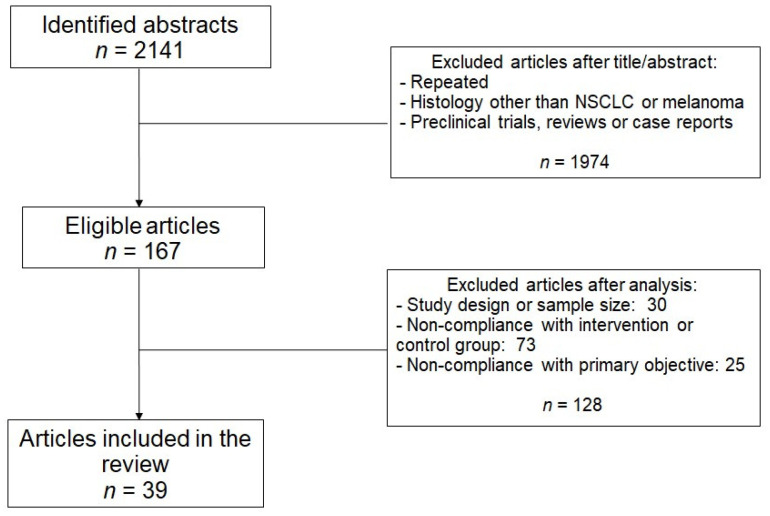
Selection of articles included in the review.

After the search, 2141 articles were identified, of which 39 articles met the criteria initially established and were selected to carry out this review. The flowchart that explains the screening process is shown in Figure 1.

In the tables presented below, the results are separated according to the target location of the radiation therapy, that is, according to whether the local treatment with SBRT/SRS was performed on cerebral or extracerebral metastases.

It is differentiated in this way by the difficulty of the immune cells to cross the blood–brain barrier after being stimulated by a local treatment with SRS in brain lesions. The difficulty of extravasating the blood–brain barrier could lead to a potential inequality in the systemic effect produced by the brain SRS compared to that produced by the SBRT on extracerebral lesions.

Abscopal effect was evaluated as a local response in non-irradiated lesions.

In both cases (cerebral and extracerebral metastases), ICI treatment regimens were administered according to the approved clinical protocol of studies with different doses: Ipilimumab (anti-CTLA4) 3 mg/kg or 10 mg/kg every 3 weeks, Pembrolizumab (anti-PD-1) 2 mg/kg, 3 mg/kg or 10 mg/kg every 3 weeks, or Nivolumab (anti-PD-1) 2 mg/kg or 3 mg/kg every 2 weeks. Some studies did not report the dose that they used.

Table 1 presents the results of the selected articles of the ICI + SRS combination in cerebral metastases.

Table 2 presents the results of the selected articles of the ICI + SBRT/SABR combination in extracerebral metastases.

## 3. Discussion

### 3.1. ICI + SRS: Cerebral Metastases

Patients with non-small-cell lung carcinoma and melanoma have a high incidence of cerebral metastases both at diagnosis and throughout the course of the disease. An incidence of cerebral metastases >25% has been observed in both tumors [53,54]. As we know, the appearance of metastases leads to a decrease in survival and, therefore, a poor prognosis in these patients [55].

Immunotherapy has shown increased survival in certain metastatic patients with NSCLC and melanoma, leading to its approval as a first-line drug in both cases [56,57]. In addition, SRS has been shown to be effective as a local treatment for cerebral metastases in patients who are candidates for this treatment [58].

Of the 25 articles included in Table 1, 21 of them included patients with metastatic melanoma [14,15,16,17,18,19,20,21,22,23,24,25,26,27,28,29,30,31,32,33,34], 2 with metastatic NSCLC [35,36], and 2 with heterogeneous histology (>80% patients with metastatic melanoma or NSCLC) [37,38].

All articles included were retrospective, except for one that was prospective [23].

The studies sample size varied significantly, with studies ranging from 11–260 patients and with the number of lesions treated ranging from 23–793. We need to consider these differences when evaluating the results.

First of all, it must be taken into account that the consideration of concomitant treatment differed between the different studies. Most authors considered concomitant ICI-SRS as the administration of SRS within 4 weeks before or after the start of ICI [23,25,29,30,32,33,34,38]. However, other authors considered a timeframe <2 weeks [31,37] and others up to >2 months [20,24]. When local treatment was SRS exclusively (SRS-only), some authors considered patients who had not received immune checkpoint inhibitors [14,16,22]. Nevertheless, other authors considered treatment with SRS exclusively when the last dose was applied at least 3 months [23] or 6 months before [27].

According to the results obtained, local control (LC) after SRS-only of cerebral lesions is 45–92.3%. On the other hand, the combination of ICI-SRS treatment places local control between 16.5% and 100%. Typically, the LC obtained in most articles with the ICI-SRS combination is greater than 70%, with the exception of the study by Cohen-Inbar et al. [25], who presented lower LC rates in their results.

If we focus exclusively on those studies that include a comparison of the treatment with SRS-only versus ICI-SRS, only two of them showed a significantly greater LC with the combination treatment [27,33], while in five others, there were no differences between these [14,16,22,29,37].

If we consider the systemic treatment administered (anti-PD-1 vs. anti-CTLA4), a higher LC was observed in patients with melanoma when anti-PD-1 was used with rates of 80–96% [17,20,27,28,34] versus anti-CTLA4 with rates of 16.5–100% [14,15,16,25,29]. Minniti G. et al. reported a statistically significant increase in LC when anti-PD-1 versus anti-CTLA4 was employed (85% vs. 70%, respectively) [31].

If we evaluate the treatment sequence (concomitant vs. sequential), the benefit in LC is more controversial. In those studies that included a comparison of the treatment sequence, in three of them, there were no significant differences [15,29,38]. However, in the study by Chen L. et al., a tendency to significance in favor of concomitant versus sequential treatment was observed (88% vs. 79% respectively; *p* = 0.08) [37]. Finally, Cohen-Inbar O. et al. did observe a statistically significant higher LC in patients treated with concomitant versus sequential SRS + ICI (54.4% vs. 16.5%; *p* < 0.05) [25]. Regarding the abscopal effect, none of the included studies on cerebral metastases reported rates of possible distant effects on non-irradiated lesions. Only Kiess et al. reported a possible patient with an abscopal response [15].

Regarding other secondary variables outside the scope of this review, some authors observed greater overall survival with the combination of ICI-SRS treatment versus treatment with SRS-only [22,29], as well as with its concomitant vs. sequential administration/SRS-only [15,16,25,29,37].

As a secondary analysis, regarding progression-free survival (PFS), the combination of ICI-SRS treatment versus SRS-only treatment also appears to show benefits [24,30,32,35]. In terms of toxicity rates, G3-G4 toxicity ranges from 5–24%. G5 toxicity was not reported in any of the studies.

There are currently several ongoing clinical trials, such as the MIGRAINE trial (NCT04427228) and STICk-IM-NSCLC (NCT04650490), that will provide more data in relation to the ICI-SRS combination treatment.

### 3.2. ICI + SBRT/SABR: Extracerebral Metastases

As mentioned above, SBRT/SABR provides a benefit in the treatment of metastatic patients [9,59].

Following the search, 13 articles that met the stated search criteria were included (Table 2). Of them, six were phase I/II clinical trials, one was a prospective study, and six were retrospective studies. The number of patients varied from 13 to 151 patients. If we consider histology, five included melanoma patients exclusively [39,40,41,42,43], three included NSCLC patients [44,45,46], and six included patients with multiple histologies [47,48,49,50,51,52]. The fractioning schemes used varied between the different studies, with multiple fractioning (3 to 10 fractions) being more frequent [39,40,41,42,43,44,45,46,47,48,49,52] compared to the single fraction [50,51].

Local control of combination treatment with SBRT/SABR + ICI ranged from 40% to 94%. However, we must take into account that the rates with an LC < 60% belong to retrospective studies and with few patients (*n* < 20) [39,47]. If we consider the results obtained in clinical trials, the LC increases to 75%–91% [41,42,48,49].

The response rate in non-irradiated lesions (abscopal effect) ranged from 1.3% [43] to 45% [42]. If we analyze the six clinical trials included, abscopal response rates between 10% and 45% are reported. Sundahl N. et al. obtained up to 45% of responses in non-irradiated lesions in melanoma patients, of which a full response was observed in 15% [42]. Welsh J.W. et al. observed an overall response of 26% in non-irradiated lesions, obtaining a greater response in lesions that incidentally received low doses of radiation compared to those that did not (31% vs. 5%; *p* < 0.05) [52]. This finding was also evidenced by Menon H. et al. in their post hoc analysis, reporting a greater response in non-irradiated lesions when receiving low doses of RT [60].

The included studies show heterogeneity in the location of the target or treatment location. In the majority of these studies, patients were compared with lesions treated in multiple locations (lung, liver, bone) that hinder an analysis of the LC or abscopal effect according to the location. Tang et al. suggested that hepatic SBRT may be associated with higher immune systemic activation than lung SBRT, given an early increase in peripheral CD8+T lymphocytes and higher PD-1 expression in CD8+T lymphocytes [48]. Luke et al. did not report differences according to the treated target but did observe a correlation between genes associated with IFN-γ expression and a greater abscopal response [49].

Four of the studies [39,40,43,51] included patients treated with SRS/cerebral radiosurgery. The study by Mowery Y. et al. [43] is the only one to report an abscopal response in a single patient when receiving cerebral SRS.

Regarding the administered systemic treatment, only one study in NSCLC evaluated differences in treatment with anti-PD-1 versus anti-CTLA4 [46]. In this study, a greater abscopal response (37% vs. 24%), overall survival (NA vs. 10.7 months), and disease-free survival (NA vs. 6.4 months) were observed in favor of treatment with anti-PD-1 in a significant way.

Based on the treatment sequence, none of the articles studied the differences in local control or abscopal effect with concomitant versus sequential ICI administration.

Finally, for toxicity ≥G3, the rate ranged from 0 to 34%.

Despite the limitations, the included clinical trials showed a high LC and an abscopal response rate (>10%) that is not inconsiderable. However, clinical trials with a larger number of patients are necessary, in which the impact of the possible abscopal effect is evaluated according to the location of treatment (in the literature, differences in immune signaling according to the irradiated organ are reported) [48,52] and the best treatment sequence.

## 4. Materials and Methods

### 4.1. ICI-SBRT/SRS Hypothesis

This systematic review aimed to evaluate the local control and systemic effect of combined ICI and stereotactic radiation therapy (SRS/SBRT) treatment in metastatic patients with NSCLC and melanoma. Overall survival (OS), progression-free survival (PFS), and toxicity of combination therapy were collected.

### 4.2. Search Strategy

The publications of the last 10 years were reviewed in the MEDLINE database (via PubMed) from January 2010 to December 2020. Articles in English were obtained whose object of study was the combination of stereotactic radiation therapy with immune checkpoints inhibitors in metastatic patients with non-small-cell lung cancer (NSCLC) and melanoma. Multiple terms were used for the search, including “Immunotherapy”, “Anti-PD1”, “Anti-PD-L1”, “Anti-CTLA4”, “Immune checkpoint inhibitors”, “Abscopal effect” and their combination with each of the following terms “SBRT”, “SABR”, “SRS”, “Radiosurgery”, “Stereotactic ablative radiation therapy”, “radiation therapy”, “non-small-cell lung cancer”, and “melanoma”. Non-original articles were excluded.

### 4.3. Selection Criteria

All articles were evaluated in a first phase according to the title and/or abstract. The articles included in the review had to be based on and comply with the previously defined PICO methodology:

(a) Metastatic patients of melanoma or non-small-cell lung cancer,
(b) Patients treated with concomitant/sequential SRS/SBRT to treatment with immune checkpoint inhibitors (anti-PD-L1, anti-PD-1, anti-CTLA4),
(c) Control group studies (patients treated with ICI without radiation therapy or with SRS/SBRT without ICI) or without a control group,
(d) Studies whose primary objective was to analyze local control and/or systemic effect (abscopal),
(e) Clinical trials, prospective studies, and retrospective studies were included.

### 4.4. Exclusion Criteria

Articles that did not meet the proper design or with a low sample size were excluded. For this purpose, the following were considered as exclusion criteria:

(a) Opinion articles, case reports, and studies with a sample size (*n*) less than 10 patients,
(b) Preclinical articles: tests with murine and in vitro models.

## 5. Conclusions

The heterogeneity in the number and histology of patients included, in the sequence and systemic treatment administered, as well as the lack of clinical trials makes it difficult to draw robust conclusions.

The combined treatment with ICI-SBRT demonstrates high local control and non-negligible abscopal response in patients with extracerebral metastases of NSCLC and melanoma with an acceptable toxicity.

However, the benefit in local control of the ICI-SRS combination in patients with cerebral metastases is more controversial. Greater local control with anti-PD-1 versus anti-CTLA-4 was observed in cerebral metastases from melanoma patients. An abscopal effect was not reported in the included studies.

Clinical trials with a larger number of patients and more homogeneous samples are needed to obtain conclusive data. Searching for predictive markers of abscopal response in combination therapy could optimize the best sequence and treatment for these patients.

## Figures and Tables

**Table 1:** Results of the ICI + SRS combination in cerebral metastases in melanoma/non-small-cell lung cancer (*n* = 25).

Author	Study Type	*n*	Nº Lesions	Median Follow up (Months)	Histology	Target	Doses/Fraction (Gy/fx)	IT	Groups	Local Control (CR + PR + SR)	Abscopal Responses	Median Survival (Months)	Median PFS (Months)	Toxicity ≥ Grade 3 (%)
Mathew M., et al., 2013 [14]	Retrospective	58	198	6	Melanoma	Brain	15–20 Gy/1fx	Anti-CTLA4	SRS SRS + IT	65% 63%	NR	5.9	NR	NR
Kiess, A.P., et al., 2015 [15]	Retrospective	46	113	NR	Melanoma	Brain	15–24 Gy/1fx	Anti-CTLA4	SRS + IT before SRS + IT conc SRS + IT after	87% 100% 89%	NR	NR	NR	G3–4: 20% G5: 0%
Patel K., et al., 2015 [16]	Retrospective	54	NR	7.3	Melanoma	Brain	NR	Anti-CTLA4	SRS SRS + IT	92.3% 71.4%	NR	NR	4.2 3.1	15%
Ahmed, et al., 2015 [17]	Retrospective	26	73	9.4	Melanoma	Brain	18–24 Gy/1fx 25–30 Gy/5fx	Anti-PD-1	SRS + IT	89%	NR	12	4.6	0%
Ahmed, et al., 2016 [18]	Retrospective	96	314	7.4	Melanoma	Brain	15–24 Gy/1fx	Various	SRS + IT	83%	NR	10,5	3.4	NR
Kotecha R., et al., 2018 [19]	Retrospective	191	793	7	Melanoma	Brain	NR	Various	SRS + IT	86%	NR	NR	NR	NR
Anderson E., et al., 2017 [20]	Retrospective	11	23	9.2	Melanoma	Brain	18–21 Gy/1fx 30Gy/5fx	Anti-PD-1	SRS + IT conc	96%	NR	ND	ND	G3: 5% G4–5: 0%
Choong E., et al., 2017 [21]	Retrospective	108	NR	8.6	Melanoma	Brain	NR	Various	SRS + IT	78%	NR	14.2	ND	3%
Kaidar-Person O., et al., 2017 [22]	Retrospective	58	NR	21	Melanoma	Brain	18–20 Gy/1fx 21–30 Gy/3–5fx	Anti-PD-1 Anti-CTLA4	SRS SRS + IT	86% 52%	NR	5.5 15	8 5	NR
Yusuf M., et al., 2017 [23]	Prospective	51	167	7	Melanoma	Brain	13–24 Gy/1fx	Anti-PD-1 Anti-CTLA4	SRS SRS + IT conc	75%	NR	NR	NR	NR
An Y., et al., 2017 [24]	Retrospective	71	257	15.5	Melanoma	Brain	16–24Gy/1fx	Anti-PD-1 Anti-CTLA4 Inhibitor BRAF	SRS + IT	90%	NR	12	NR	NR
Cohen-Inbar O., et al., 2017 [25]	Retrospective	46	232	7.9	Melanoma	Brain	14–22 Gy/1fx	Anti-CTLA4	SRS + IT conc SRS + IT seq	54.4% 16.5%	NR	13.8 6.4	7.2 5	NR
Robin T., et al., 2018 [26]	Retrospective	38	NR	31.6	Melanoma	Brain	NR	Anti-PD-1 Anti-CTLA4	SRS + IT	92%	NR	NA	3.4	G3: 8% G4–5: 0%
Trommer M., et al., 2018 [27]	Retrospective	26	48	NR	Melanoma	Brain	18–22 Gy/1fx	Anti-PD-1	SRS + IT SRS	86% 80%	NR	NR	NR	0%
**Author**	**Study Type**	***n***	**Nº Lesions**	**Median Follow up** **(Months)**	**Histology**	**Target**	**Doses/Fraction**	**IT**	**Groups**	**Local Control** **(CR + PR + SR)**	**Abscopal** **Responses**	**Median Survival** **(Months)**	**Median PFS** **(Months)**	**Toxicity ≥ Grade 3 (%)**
Nardin C., et al., 2018 [28]	Retrospective	74	NR	14	Melanoma	Brain	12–24 Gy/1fx 24–35 Gy/1–5fx	Anti-PD-1	SRS + IT	80%	NR	15.3	3	G3: 12% G4–5: 0%
Diao K., et al., 2018 [29]	Retrospective	91	NR	NR	Melanoma	Brain	12–22 Gy/1fx	Anti-CTLA4	SRS SRS + IT conc SRS + IT seq	45% 60% 70%	NR	7.8 11.8 18.7	NR	G3–4: 5% G5: 0%
Stera S., et al., 2018 [30]	Retrospective	45	250	8,3	Melanoma	Brain	15–20 Gy/1fx	Anti-PD-1 Anti-CTLA4 Inh BRAF/MEK	SRS + IT	89.5%	NR	NR	NR	G3: 8.33% G4–5:0%
Minniti G., et al., 2019 [31]	Retrospective	80	326	15	Melanoma	Brain	18–22 Gy/1fx 27 Gy/3fx	Anti-PD-1 Anti-CTLA4	SRS + anti-PD1 SRS+anti-CTLA4	85% 70%	NR	22 14.7	NR	24% (G3) 17% (G3)
Murphy B., et al., 2019 [32]	Retrospective	26	90	18.9	Melanoma	Brain	22 Gy/1fx 27–30 Gy/3–5 fx	Anti-PD-1 Anti-CTLA4	SRS + IT conc SRS + IT seq	95.4%	NR	26.1	19 3.4	G3: 8% G4–5:0%
Hadi I., et al., 2020 [33]	Retrospective	30	52	19	Melanoma	Brain	18-24 Gy /1fx	Various	SRS + IT SRS	100% 83.3%	NR	22	16	13.5%
Carron R., et al., 2020 [34]	Retrospective	50	181	38.9	Melanoma	Brain	22–26 Gy/1fx	Anti-PD-1	SRS + IT	94%	NR	16.6	13.2	14.6%
Ahmed K., et al., 2017 [35]	Retrospective	17	49	8.6	NSCLC	Brain	18–24 Gy/1fx 25 Gy/5fx	Anti-PD-1 Anti- PD-L1	SRS + IT	96%	NR	5.7	NR	NR
Singh C., et al., 2019 [36]	Retrospective	85	531	12	NSCLC	Brain	12–24 Gy/1–3 fx	Anti-PD-1 +/- anti-CTLA4	SRS + IT SRS + QT	97% 96.6%	NR	10 11.6	4.6 6.1	NR
Chen L., et al., 2018 [37]	Retrospective	260	623	9.2	NSCLC (60%) Melanoma (30%) Renal (10%)	Brain	15–24 Gy/1fx 18–24 Gy/3fx 25 Gy/5fx	Anti-PD-1 Anti-CTLA4	SRS SRS+ IT seq SRS +IT conc	82% 79% 88%	NR	12.9 14.5 24.7	NR	G3: 19% G4: 1% G5: 0%
Koening J., et al., 2019 [38]	Retrospective	97	580	NR	Melanoma (39%) NSCLC (46%) Others (15%)	Brain	22–30 Gy/1–5 fx	Anti-PD-1 Anti- PD-L1 Anti-CTLA4	SRS+ IT conc SRS+IT seq	96% 97%	NR	9.4	NR	G3: 7% G4: 7%

IT: Immunotherapy; CR: Complete response; PR: Partial response; SR: Stable response; NR: Not reported; Conc: Concomitant; Seq: Sequential.

**Table 2:** Results of the ICI + SRS combination in extracerebral metastases in melanoma/non-small-cell lung cancer (*n* = 14).

Author	Study Type	*n*	Median Follow up (Months)	Histology	Target	Doses/Fraction (Gy/fx)	IT	Groups	Local Control (CR + PR + SR)	Abscopal Responses	Median Survival (Months)	Median PFS (Months)	Toxicity ≥ Grade 3 (%)
Kropp L., et al., 2016 [39]	Retrospective	16	25.5	Melanoma	Visceral 11% Brain	30 Gy/5fx 36 Gy/6fx	Anti-CTLA4	SBRT + IT seq	56%	NR	NR	NR	0%
Koller K., et al., 2017 [40]	Retrospective	101	19	Melanoma	Visceral Bone Brain	NR (13% SBRT)	Anti-CTLA4	RT + IT conc IT	NR	37.1%	19 10	5 3	NR
Sundahl N., et al., 2018 [41]	Phase I	13	NR	Melanoma	Visceral Bone	24–36 Gy/3 fx	Anti-CTLA4	SBRT+IT conc	91%	23%	18.5	NR	25%
Sundah N., et al., 2019 [42]	Phase II	20	13.1	Melanoma	Visceral	8 Gy/3 fx	Anti-PD-1	SBRT+IT conc	90%	45%	NR	NR	G3: 15% G4–5: 0%
Mowery Y., et al., 2019 [43]	Retrospective	151	12.9	Melanoma	Various	NR	Anti-PD-1	RT + IT (SRS 26%) IT	NR	1.31%	NR	5 NA	G3: NR G4–5: 0%
Lesueur P., et al., 2018 [44]	Retrospective	104	15.8	NSCLC	Visceral Bone Brain	SBRT: 20–36 Gy/1–6fx RT: 20–30Gy/5–10fx	Anti-PD-1	RT/SBRT + IT	64.4%	NR	11	2.7	G3–4: 14.4% G5: 0%
Formenti S., et al., 2018 [45]	Prospective	39	43	NSCLC	Visceral Bone	30 Gy/5fx or 27 Gy/3fx	Anti-CTLA4	SBRT+ IT seq	NR	18%	7.4	3.81	0%
Chen L., et al., 2020 [46]	Retrospective	33	NR	NSCLC	Visceral	60 Gy/10fx or 50 Gy/4fx	Anti-PD-1 Anti-CTLA4	SBRT+anti-PD-1 SBRT +anti-CTLA4	88% 94%	37% 24%	NA 10.7	NA 6.4	19% 29%
Ribeiro Gomes J., et al., 2016 [47]	Retrospective	16	8	Melanoma (75%) NSCLC (12.5%) Renal (12.5%)	Visceral Bone	24 Gy (1–40)/ 3fx (1–10)	Anti-PD-1	SBRT+ IT	40%	18.7%	7.4	NR	0%
Tang C., et al., 2017 [48]	Phase I	35	9.3	Various (NSCLC 22.8%)	Visceral	Conc: 50 Gy/4fx Seq: 50 Gy/4fx or 60 Gy/10fx	Anti-CTLA4	SBRT conc SBRT seq	90.3%	10%	10.2	3.2	G3: 34% G4–5: 0%
Luke J., et al., 2018 [49]	Phase I	79	7.1	Various (NSCLC 9.6%)	Visceral	30–50 Gy/3–5 fx	Anti-PD-1	SBRT + IT seq	75%	13.5%	9.6	3.1	9,6%
Maity A., et al., 2018 [50]	Phase I	24	NR	Various Melanoma (17%) NSCLC (33%)	Visceral Bone	24 Gy/3fx or 17 Gy/1fx	Anti-PD-1	SBRT+IT conc	NR	12.5%	6.9	1.9	G3: 33% G4–5: 0%
Desideri I., et al., 2018 [51]	Retrospective	20	NR	NSCLC (85%) Renal (15%)	Visceral Brain	SBRT:18–40 Gy/1–5fx RT: 8–30 Gy/1–10fx	Anti-PD-1	SBRT+ IT conc RT + IT conc	87.5% NR	NR	17.9 10.3	11.5 5.2	G3: 15% G4–5: 0%
Welsh J., et al., 2019 [52]	Phase II	106	10.5	Various (NSCL 18%)	Visceral	60 Gy/10fx 50 Gy/4fx	Anti-CTLA4	SBRT+IT seq SBRT + IT conc	NR	26%	NA	2.9	G3: 33% G4–5: 0%

## Data Availability

Not applicable.

## Abbreviations

RT	Radiotherapy
SRS	Stereotactic radiosurgery
SABR	Stereotactic ablative body radiotherapy
PD-1	Programmed death 1
NSCLC	Non-small-cell lung cancer
BED	Biological effective dose
ICI	Immune checkpoints inhibitors
SBRT	Stereotactic body radiotherapy
CTLA-4	Cytotoxic T-Lymphocyte Antigen 4.
PD-L1	Programmed death ligand 1
BM	Brain metastases
